# The lncRNA Toolkit: Databases and In Silico Tools for lncRNA Analysis

**DOI:** 10.3390/ncrna6040049

**Published:** 2020-12-16

**Authors:** Holly R. Pinkney, Brandon M. Wright, Sarah D. Diermeier

**Affiliations:** Department of Biochemistry, University of Otago, Dunedin 9016, New Zealand; holly.pinkney@postgrad.otago.ac.nz (H.R.P.); wribr677@student.otago.ac.nz (B.M.W.)

**Keywords:** non-coding RNAs, long non-coding RNAs, databases, computational analysis, bioinformatic prediction software, RNA interactions, coding potential, RNA structure, RNA function

## Abstract

Long non-coding RNAs (lncRNAs) are a rapidly expanding field of research, with many new transcripts identified each year. However, only a small subset of lncRNAs has been characterized functionally thus far. To aid investigating the mechanisms of action by which new lncRNAs act, bioinformatic tools and databases are invaluable. Here, we review a selection of computational tools and databases for the in silico analysis of lncRNAs, including tissue-specific expression, protein coding potential, subcellular localization, structural conformation, and interaction partners. The assembled lncRNA toolkit is aimed primarily at experimental researchers as a useful starting point to guide wet-lab experiments, mainly containing multi-functional, user-friendly interfaces. With more and more new lncRNA analysis tools available, it will be essential to provide continuous updates and maintain the availability of key software in the future.

## 1. Introduction

**Figure 1:**
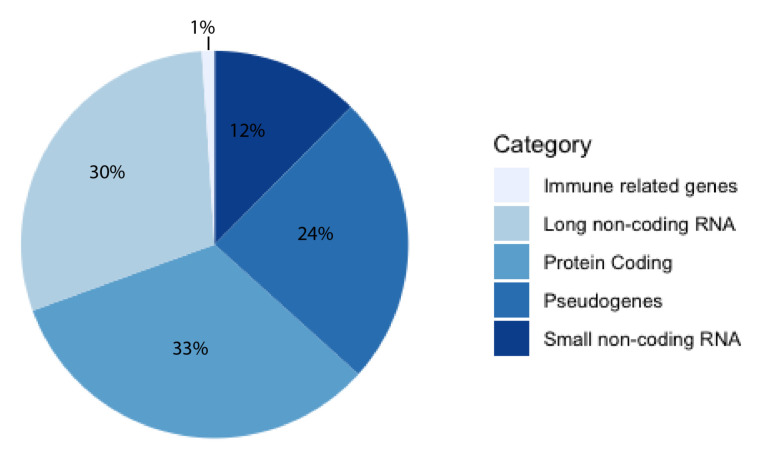
Pie chart of gene categories in the human genome. Data taken from GENCODE release 35.

For decades, the human genome was thought to be a desert of ‘junk DNA’ with sporadic oases of transcriptionally active genes, most of them coding for proteins. This theory has since fallen out of favor with the growing support of pervasive transcription [1]. The human genome is now thought to be more akin to a jungle, where great swathes are transcriptionally active and interacting with other constituents, forming a delicate milieu of reciprocity [2]. The majority of known human genes are non-coding [3], and approximately half of these are long non-coding RNAs (lncRNAs), according to the latest release of GENCODE (Figure 1). Estimates of the exact number of lncRNA genes vary, with high numbers of up to 100,000 genes [4]. A lncRNA is a non-coding transcript longer than 200 nucleotides (nt) comprising both RNA polymerase II and III transcripts, with the former often being spliced, capped, and polyadenylated [5]. LncRNAs have been shown to serve a myriad of functions, from X-chromosome inactivation [6] to regulation of gene expression on the chromatin level [7] and post-transcriptional regulation [8]. They have been implicated as drivers of numerous diseases, including cancers [9,10,11], Alzheimer’s disease [12], inflammatory bowel disease [13], autoimmune disease [14] and diabetes [15]. Although there are several well-characterized lncRNAs, there are currently orders of magnitude more that are poorly understood. This illustrates the need for new predictive tools and databases to aid in their study. Computational tools can be useful for researchers aiming to investigate a newly discovered and previously uncharacterized lncRNA, as obtained in silico results may be helpful in guiding experimental set-up. For example, if a localization predictor indicated that a lncRNA is nuclear-retained, and an interaction predictor returned DNA-binding motifs, investigating the potential of gene expression regulation may be a useful starting point for the lncRNA of interest.

In this review, we detail a selection of lncRNA predictive tools and databases with the aim of providing a toolkit for experimental scientists. Although numerous tools and databases are available, with new ones being added frequently, we chose the tools discussed here based on accuracy, functionality, frequency of updates, and ease of use for biologists. Links to all included tools and databases are included in Table A1.

## 2. General lncRNA Databases

**Figure 2:**
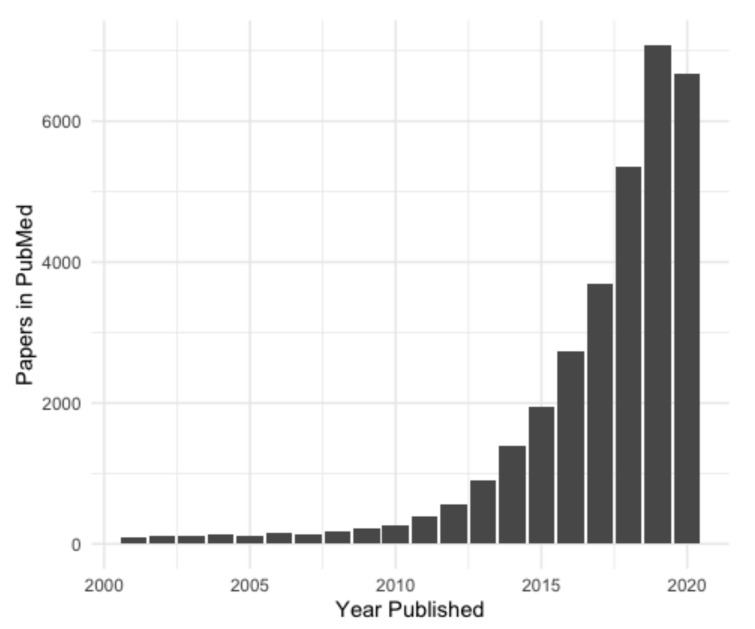
Number of lncRNA papers published from 2001. Identified by keyword, accessed from PubMed October 2020.

In recent years, there has been an exponential increase in lncRNA research (Figure 2) and the number of new non-coding transcripts being identified [5]. Accurate and easily accessible databases are needed to curate this influx of putative lncRNA genes, especially considering that the identification of new lncRNA genes is usually based on RNA-sequencing (RNA-seq), and every transcript annotated as lncRNA may not, in fact, be one. Here we will discuss two current ‘meta-databases’ of lncRNAs, which integrate data from a variety of sources. These were chosen as they are well curated, collate data from many sources and integrate them in an easily accessible format. Numerous other databases of lncRNAs are available; however, most of these are subsets of the databases covered here (NONCODE, FANTOM CAT, etc.), or were constructed for a specific purpose, such as for a particular disease (Lnc2Cancer [16]) or species (GreeNC [17]).

### 2.1. LNCipedia

Originally released in 2013 [18], the latest release (v5) of LNCipedia was published in 2019 [19]. LNCipedia contains data from ten different sources as of 2019: LncRNAdb version 1 [20], the Broad Institute human body map of lincRNAs [21], Ensembl release 92 [22], RefSeq 106 [23], NONCODE v4 [4], FANTOM CAT (stringent set, a version of the database with more strict entry criteria) [24] and three research papers: Hangauer et al. 2013 [1], Nielsen et al. 2014 [25], and Sun et al. 2015 [26]. The dataset was aggregated to remove low confidence lncRNAs with less than two transcripts each from a different source matching perfectly at each exon [19]. In addition, transcripts that did not map to the current hg38 reference genome, were shorter than 200 nucleotides, or contained exons overlapping with coding sequences were filtered out [19]. After aggregation and filtering, LNCipedia v5 contains 127,802 transcripts from 56,946 genes in the full dataset [19]. The high-confidence set, which showed no coding potential using coding prediction tools (covered in Section 4 of this review) , contained 10% fewer lncRNAs than the full set [19]. LNCipedia represents one of the largest lncRNA databases to date. Since its initial release, LNCipedia has received major updates with extensive improvements in the database itself as well as to the user interface [19]. Consistent updating is crucial for a lncRNA database, as multitudes of novel lncRNA transcripts are identified each year. LNCipedia offers a well-designed and intuitive web-based interface, with the option to download the database for offline accession. LncRNAs can be searched using their transcript name or Gene ID. The results page provides data including the sequence, known isoforms, coding potential, locus conservation across a few species, and any published literature. A potential drawback of LNCipedia is the stringent filtering criteria. Considering that many lncRNAs such as antisense transcripts overlap with protein coding genes [27], this may have resulted in numerous biologically relevant lncRNAs being omitted from the database. Although LNCipedia offers built-in prediction of coding potential (as discussed in Section 4 of this review) there is no automated prediction of subcellular localization, association with disease or functional prediction.

### 2.2. LNCBook

In contrast to LNCipedia, LNCBook collates lncRNAs from both pre-existing databases and experimentally verified community-curated transcripts [28]. The pre-existing databases include GENCODE v27 [29], NONCODE v5.0 [4], and LNCipedia v4.1 [19]. LNCipedia was used in addition to NONCODE and other databases within LNCipedia to ensure that any lncRNAs omitted from LNCipedia during filtering were still included in LNCBook. The community-curated transcripts are derived from LncRNAWiki, a community driven database created by the authors of LNCBook in 2015 [30]. This MediaWiki-based database allows users to upload lncRNAs identified in their own research [28]. From both sources a total of 268,848 transcripts were identified from 140,356 non-redundant lncRNA genes [28]. LNCBook offers multi-omics integration, such as expression profiles across tissue types (both normal and cancer), DNA methylation patterns in different gene regions, genomic variation and microRNA (miRNA) interaction predictions using TargetScan [31] and miRanda [28,32]. Similar to LNCipedia, LNCBook offers built-in coding potential prediction through CPAT [33] and PLEK [34], which are discussed later in this review in Section 4 [28]. Although LNCBook provides an extensive amount of information for each lncRNA, it has not received a major update since version 1 since its initial release in October 2018. As frequent updating is one of the most important features of databases, it remains to be seen whether LNCBook will become the new standard of lncRNA databases.

Both general lncRNA databases covered here offer an extensive list of long non-coding transcripts, in addition to intuitive interfaces, integrated literature searches and conservation across species. Future improvements for these and other databases could include further integration of predictive tools and additional experimental data, such as subcellular localization, and interaction partners of lncRNAs. Although no comprehensive review of lncRNA databases has been published recently, there are a multitude of different databases available, ranging from general to highly specific. These include but are not limited to: MONOCLdb for mouse lung lncRNAs [35], the Cancer lncRNA Census [36] and GreenNC for plant lncRNAs [17]. LncRNAs often have several different identifiers, including gene IDs, HUGO gene names, ENSEMBL identifiers, ENTREZ gene IDs and many more. This can make it difficult to find information on a specific lncRNA when only one identifier is known, or when a database uses a separate set of identifiers. A comprehensive list of identifiers for each lncRNA, or overall more standardized nomenclature, would be useful for the field.

## 3. LncRNA Expression Databases

LncRNAs are usually expressed in a cell-type and tissue-specific manner [37], indicating their importance in developmental processes and disease mechanisms [38]. When characterizing a new lncRNA, expression databases compiling numerous RNA-seq experiments are a useful starting point to elucidate the function. Here, we will discuss two well-known expression databases for human tissue, and one for lncRNA expression in plants.

### 3.1. GTEx

The Genotype-Tissue Expression database (GTEx) is a project first described in 2013 by the Broad Institute [39]. It is being updated continuously, with a major update in September 2020 (GTEx v8) [40]. Although originally conceived as an expression database, GTEx version 8.2 (the most recent release at the time of writing) contains high-throughput sequencing data from 948 human subjects, spanning 54 different tissues, with RNA-, DNA-, and CHIP-seq (Chromatin Immunoprecipitation-sequencing) data for at least 70 samples per tissue [40]. In addition to comprehensive expression data from 17,382 RNA-seq experiments, GTEx also contains whole genome sequencing data to a depth of 32x coverage, providing an extensive inventory of genetic, epigenetic and splicing variants [40]. Information on disease associations and context specific genetic effects, and Quantitative Trait Locus (QTL) information is available, further allowing insight into genomic loci which affect expression (eQTL data) or splicing (sQTL data) of both protein coding and long non-coding genes [40]. GTEx has compiled datasets available for download, and histology images categorized by tissue type. It even provides access to biospecimens upon application. Although GTEx is a useful resource for the scientific community, it should be noted that out of a total of 17,382 RNA samples, 42% (7251 samples) showed an RNA integrity number (RIN) value between 5.5 and 6.5, where 7 is usually the minimum quality cutoff for an RNA sample [41]. Additionally, race, gender and age are not equally represented across all samples, which may confound the data to some degree.

### 3.2. TANRIC

The Atlas of Non-coding RNAs in Cancer (TANRIC) was first compiled in 2015 [42]. The database is designed specifically to explore the genomic and clinical relevance of lncRNAs in cancer. TANRIC v2.0 was released online only in late 2019, expanding the database content to cover over 40 different cancer types and cell lineages. This information was taken from The Cancer Genome Atlas (TCGA) [43], the Cancer Cell Line Encyclopedia (CCLE) [44], and from three additional independent cancer cohorts [45,46,47]. For some tumor samples, RNA-seq information is available for matched metastases and normal tissue as well. Expression levels were quantified using RPKM, and clinical metadata (such as staging, grade, subtype and survival) have been included, along with molecular characteristics of the cancer (for example, HER2 receptor or microsatellite instability status). TANRIC is a useful tool to explore cancer-specific expression of any given lncRNA. The results can be inspected in the context of clinical information, such as the automatically generated correlation with patient survival, or expression across different cancer subtypes. TANRIC also outputs correlation information for somatic copy number variation, and mutations, as well as associated mRNAs, miRNAs and proteins.

### 3.3. CANTATAdb

CANTATAdb is a lncRNA database specifically for plants [48]. It was first released in 2016, containing 45,117 lncRNAs from ten different model plant species, including *A. thaliana*, *B. rapa*, *G. max* and *M. domestica*. This has since been updated to CANTATAdb v2.0 in 2019, increasing the database content to 239,631 lncRNAs across 39 different plant species (three of which are algal species) [48]. LncRNAs were manually curated from paired-end RNA-seq data from 328 separate experiments [49]. CANTATAdb can be queried by gene ID for a particular species, and outputs the resulting lncRNA sequence, genomic location, coding potential status prediction, information on expression across RNA-seq libraries and any matches against transcripts present in the Basic Local Alignment Search Tool (BLAST) [50] and NONCODE [4] databases. Overall, CANTATAdb is a well compiled database of great value for plant biologists.

An emerging field in lncRNA expression databases is the collation of single-cell expression profiles. This allows researchers to understand the transcriptomes of single cells, as opposed to the average expression across a tissue as a whole [51]. What lies on the horizon for this field is the release of a complete atlas of human single-cell transcriptomes, which would offer an unprecedented glimpse into truly cell-type specific expression across the human body [52,53].

## 4. Protein Coding Potential

Identifying the coding potential of a novel transcript is the vital first step in accurate annotation and downstream analysis of a lncRNA [54]. Determining coding potential through in vitro methodologies can be time consuming and resource intensive, especially when assaying multiple transcripts simultaneously. The in silico approaches described here provide a viable starting point in determining the localization of a lncRNA. In recent years, a number of predictive tools have been released that are able to determine the probability of a transcript producing a protein, based on the sequence alone. One of the earliest established tools is CPC (Coding Potential Calculator) [54], which was first released in 2007. CPC uses six features in its predictive model, including coverage of the predicted open reading frame (ORF), and sequence similarity to known protein coding genes [54]. These features were incorporated into an SVM (Support Vector Machine) machine learning classifier [54]. Although CPC was very well received at its release, more accurate tools have been published since. Detailed here are two tools used in predicting the coding potential of a transcript, chosen based on their ease of use and accuracy.

### 4.1. CPPred

Recent studies revealed that some transcripts previously classified as lncRNAs encode micropeptides [55,56,57]. Micropeptides are polypeptides with a length of <150 amino acids (aa), transcribed from short ORFs (sORFs) [58]. Many coding potential predictive tools fail to account for these micropeptides, and thus could incorrectly classify a transcript as non-coding. Building on the work on CPC by Kong et al. [54], CPPred (Coding Potential Prediction) was developed to better distinguish between transcripts encoding micropeptides, and true non-coding RNAs [59]. The sequence features chosen for machine learning in CPPred were: ORF length, ORF coverage, ORF integrity, Fickett score, Hexamer score, Isoelectric point (pI) of a predicted peptide, Grand average of hydropathicity (Gravy) of a predicted peptide, estimation of the stability of a predicted peptide and global descriptor features [59]. These ten sequence features were used in an SVM to differentiate between coding and non-coding RNAs [59]. Data for training and testing was collated from NCBI and Ensembl, for a total of 50,040 coding and 36,360 non-coding RNAs respectively [59]. The data set was split, with two thirds being used to train the model and the remainder used as a testing set [59]. To evaluate the performance of CPPred, it was benchmarked against other established predictive tools (CPAT [33], CPC2 [60] and PLEK [34]) in *H. sapiens*, *D. melanogaster*, *D. rerio*, and *S. cerevisiae*. CPPred outperformed the other tools in the non-human tests, but was less accurate than PLEK in the human set. Although most tools available for coding potential prediction are implemented in a web-server-based interface, CPPred is only available as a Python package at present, potentially making it difficult to use for those unfamiliar with programming languages.

### 4.2. CNIT

CNIT (Coding-Non-coding Identifying Tool) [61] was built on the authors’ previous predictive algorithm, CNCI (Coding-Non-Coding Index) [62], and as such uses a similar methodology [61]. First, 19,752 coding RNAs and 19,752 non-coding RNAs of human origin (GRCh38) were collected from RefSeq [23] and Ensembl [22]. Two thirds were used as a training set, and one third as a testing set [61]. Multiple features were extracted from the testing set for model construction, all based around a comparison frequency matrix of adjoining nucleotide triplets [61]. Next, the authors benchmarked the model against three other well-established predictive tools, CPC2 [60], CPAT [33] and PLEK [63], in addition to their previously published tool, CNCI [62]. The comparison was performed by using established datasets from six species: *H. sapiens*, *A. thaliana*, *M. musculus*, *D. melanogaster*, *D. rerio* and *C. elegans*. CNIT performed better than the other programs in *H. sapiens*, *A. thaliana*, and *D. rerio* [61]. CNIT was also tested with sORFs to determine its accuracy in predicting micropeptide synthesis, where it was only out competed by PLEK [61]. In addition to their animal model, CNIT also offers a model trained in plants, which greatly improves the versatility of the tool [61].

An important consideration in the development of new tools for coding potential prediction is training them to work across species, and ensure that micropeptide producing transcripts are not misclassified. The tools covered here are by no means an exhaustive list of options, with other commonly used tools including PhyloCSF [64] and COME [65]. In vitro methodologies for determining the protein coding potential of a putative lncRNA include in vitro transcription/translation assays coupled with chemiluminescent or colorimetric detection [66].

## 5. Subcellular Localization

The subcellular localization of lncRNAs is an important factor in understanding their potential function. LncRNAs localizing to the nucleus are often involved in regulating gene expression and/or splicing [67], imprinting genes [68] or inactivating the X-chromosome [6]. On the other hand, lncRNAs exported to the cytoplasm can modulate mRNA stability [69] and translation [70], regulate protein modification [71], or compete for miRNAs [72]. Databases of known lncRNA localization and tools to predict the localization of novel lncRNAs can be useful to guide experimental approaches.

### 5.1. LncSLdb

Published in 2018, LncSLdb (lncRNA subcellular localization database) is a database driven application detailing the subcellular localization data for >11,000 non-coding transcripts [73]. The data for LncSLdb was collected using two complementary approaches. First, literature available on PubMed under the key words ‘lncRNA’ and ‘subcellular localization’ was retrieved, yielding 3000 papers [73]. The list was refined to 100 papers, based on manual curation of the localization data [73]. Secondly, multiple pre-existing databases including UCSC [74], Ensembl [22], GENCODE [29] and Flybase [75] were used to gather lncRNA gene information (e.g., transcript length and genomic location) and localization data where available [73]. Each entry in the compiled database describes the subcellular localization of a lncRNA as nuclear or cytoplasmic, unless more detailed compartmental information is available. LncSLdb is a comprehensive database of lncRNA localization, with an easy-to-use web server interface.

### 5.2. LncATLAS

The LncATLAS database of lncRNA localization was developed from subcellular RNA-Seq data published by the ENCODE Consortium [3]. Data was collected from 15 human cell lines, across 48 separate experiments, comprising both adult and embryonic cell lines, for a total of 6668 genes [76]. In addition, reference genes with well documented localization (such as *MALAT1* which is nuclear-retained, or *DANCR* which predominantly localizes to the cytoplasm) can be added to the query. The localization of each lncRNA is quantified using their Relative Concentration Index (RCI), a log2-transformed ratio of respective FPKMs in the nucleus and cytoplasm. The RCI is proposed to simplify assessment of localization of a lncRNA, with a positive value indicating cytoplasmic and a negative value indicating nuclear localization.

Although databases of lncRNA localization provide accurate data across several cell lines, they are limited by the need for pre-existing experimental data, and are not suitable for lncRNAs that have not been previously captured by high-throughput experiments. Recently, new computational tools to predict subcellular localization for novel transcripts have been developed, including LncLocator [77], iLoc-lncRNa [78], and DeepLncRNA [79]. In comparison, MemPype, the first protein localization prediction tool was published a decade ago [80]. This lag in lncRNA localization prediction is likely due to lncRNAs being an emerging field, and a comparative lack of large-scale lncRNA localization data for training. Below we discuss the currently most popular tool for predicting lncRNA localization, LncLocator.

### 5.3. LncLocator

Released in 2018, LncLocator was the first tool dedicated to predicting the subcellular localization of lncRNAs [77]. The web-based software integrates two features for prediction, fed into two statistical learning models. The first of these features is raw k-mer frequency, which has been proven as a powerful feature for differentiating between lncRNAs and mRNAs [81] and lncRNA-miRNA interactions [82]. However, prediction of subcellular localization based on k-mer frequency can be challenging due to potential mutational noise [77]. To overcome this, the authors used an unsupervised stacked autoencoder to extract high-level abstractions from the sequence [77]. This is a model of an artificial neural network where the input data is encoded into a compressed representation, and then decoded into a reconstruction of the original data [83]. The two features, k-mer frequency and high-level abstractions, were each used in two prediction engines: random forest and SVM [77]. A stacked ensemble takes the prediction results from each of the four classifiers and combines them into a final decision, increasing the power of the prediction [84]. The interface will generate a probability score on a scale from 0 to 1 for each of five subcellular locales: cytoplasm, nucleus, ribosome, cytosol, and exosome, where a higher score indicates a higher probability of the lncRNA localizing to that compartment. It should be noted that LncLocator was found to achieve an accuracy of 0.59 when tested on a constructed benchmark dataset [77]. Although a useful addition to the in silico lncRNA toolkit LncLocator, such as many predictive tools, will only provide a best guess and always require experimental validations, such as RT-qPCR of fractionated cells [85] or RNA fluorescence in situ hybridization (RNA-FISH) [86].

## 6. Structural Conformation

The secondary and tertiary structure of a lncRNA may reveal information about possible interaction partners and function of the transcript, such as for the lncRNA Maternally Expressed Gene 3 (*MEG3*), which comprises three predicted structural motifs which are conserved across its multiple isoforms [87]. Two of these three motifs are required for the activation of the tumor suppressor p53, while the other is involved in suppression of DNA synthesis [87]. However, experimental investigation of lncRNA structures is challenging for several reasons. First, RNAs can change in conformation depending on their binding partners, such as in the case of the lncRNA *Braveheart*, which changes its 3D structure upon binding to Cellular Nucleic acid Binding Protein (CNBP) [88]. Secondly, lncRNA structures tend to contain dynamic regions, such as those found in the lncRNA *HOTAIR*, the structure of which was recently determined using atomic force microscopy [89]. It is challenging to determine dynamic regions of RNAs using traditional methods such as X-Ray crystallography [90]. Finally, one lncRNA gene may give rise to multiple splicing isoforms, resulting in several different corresponding RNA structures, such as the lncRNA Steroid receptor RNA activator (*SRA*) [91]. The implications of lncRNA structural conformation and function have been reviewed in detail by Chillón and Marcia (2020) [92], and Zampetaki et al. (2018) [93]. Here, we will discuss a database [94] and two prediction tools [95,96] for the investigation of 2D lncRNA structures.

### 6.1. RMDB

Determining the structural conformation of RNAs is an emerging field, with many contributing factors remaining unknown thus far [93]. As the importance of RNA structure on its function became increasingly evident recently, several experimental methods to probe RNA structure were developed, first on individual RNA level and later transcriptome-wide. These approaches include selective 2’ hydroxyl acylation with primer extension (SHAPE) and its derivatives SHAPE-Map and SHAPE-seq [97,98,99], as well as psoralen analysis of RNA interactions and structures (PARIS) [100], sequencing of psoralen crosslinked, ligated, and selected hybrids (SPLASH) [101] and Ligation of interacting RNA followed by high-throughput sequencing (LIGR-seq) [102]. With the influx of such high-throughput datasets, computational efforts to compile and share community-based structural information started alongside. This has resulted in the formation of a database designed to be the RNA equivalent of the Protein DataBank (PDB) [103] called the RNA Mapping Database (RMDB) [94]. The RMDB was first compiled in 2012 [104], before undergoing a major update in 2017 [94]. To date, it contains 769 structure entries from 148,037 RNA sequences. These are from multiple different types of experiments, including SHAPE, hydroxyl radical footprinting [105] and base methylation by dimethyl sulfate [106], among others [104]. RMDB could be further improved by the addition of structures detected by more recently developed methods such as PARIS [100]. RMDB is currently somewhat limited for lncRNA-focused biologists; however, as the number of high-throughput datasets of RNA structures is likely going to increase, the database may become more valuable for the lncRNA field in the future.

If structural information for a lncRNA of interest is not part of a database or previously generated experimental datasets, RNA structure prediction tools can be used to predict secondary structure. These tools use a variety of methods, including Minimum Free Energy (MFE)-based approaches [95] and deep learning [96]. Here, we will discuss tools from both categories, covering structure prediction tools RNAfold [95] and DMfold [96].

### 6.2. RNAfold

RNAfold is an RNA structure prediction tool from the Vienna Websuite [95]. RNAfold predicts MFE structures for single stranded sequences up to 10,000 nts in length by taking into account both binding energy and sequence accessibility, improving the likelihood of accurate results in two independent benchmarks of structure prediction tools [107,108]. RNAfold is built on Zuker and Stiegler’s dynamic programming algorithm [109], which incorporates stacking and destabilizing energies, at the same time gradually increasing the sequence length in a stepwise fashion, to predict the best structure at each increasing length. This incremental length increase allows for rapid prediction of the final, full length structure. RNAfold also uses the McCaskill partition function [110] to predict base-pairing probabilities of the structure (limited to structures of up to 7500 nts in length). Both algorithms have been adjusted by the authors to take into account the formation of circular RNA structures (circRNAs), which some lncRNAs are known to form [111]. RNAfold outputs both an optimal MFE structure prediction as well as a centroid structure, which represents the total base-pair distance to all structures that fall within the thermodynamic ensemble [95]. A high level of agreement between the MFE structure and the centroid structure indicate a more reliable prediction [95]. Both the MFE and the centroid structure outputs can be downloaded directly, or viewed using the forna visualization tool [112], which provides an interactive visualization of secondary structure. RNAfold is set up as an intuitive webserver and able to provide rapid structural predictions. However, it is limited in its ability to predict more complex structures, in particular, pseudoknots, a common structural motif [113]. These RNA structures fold back on and base-pair with itself, and are known to be challenging for many RNA structure prediction tools [113,114].

### 6.3. DMfold

DMfold is an alternative prediction tool that unlike RNAfold, can take pseudoknots into account using deep learning [96]. DMfold comprises two stages: the prediction unit and the correction unit. The prediction unit is based on a multilayered, long short-term memory, sequence to sequence deep learning model that was proposed by Sutskever et al. in 2014 [115]. The prediction unit takes FASTA formatted RNA sequences as input and makes a prediction on the secondary structure, using an encoder and decoder module to encode sequences as vectors and then decode these into secondary structure symbols. The decoder outputs a dot-bracket sequence complementary to the RNA sequence. The prediction unit was trained on a publicly available dataset of 3975 known RNA primary sequences and structures [116]. Ten percent of these sequences were set aside as a pure testing set. Once the prediction unit has generated a secondary structure prediction, the correction unit analyses it and determines if there are any errors based on stem and loop region rules. The correction unit then uses an improved base-pair maximization principle to determine optimal compatible stem regions, making corrections to the output of the prediction unit, and outputting a set of pseudoknot-free secondary structures in dot-bracket notation. These are then spliced to predict the secondary structure with pseudoknots present [96]. On the testing dataset, DMfold performed remarkably well, with positive predictive values of >0.9 (on a scale of 0 to 1, where 1 is a perfect predictor) for short sequences (70–200 nts, such as tRNA and 5s rRNA families). For longer sequences of 300–500 nts in length (such as those from transfer-messenger RNA and RNaseP families), the positive predictive value dropped to values >0.7; however, DMfold still outperformed similar tools [96]. Although DMfold is a command-line tool and therefore less accessible to non-specialist users, it excels at accuracy and pseudoknot determination. This is important for lncRNAs, which have been shown to form pseudoknot structures, such as for the conserved motifs of *MEG3* required for p53 activation [117].

Finally, RNA structure prediction in general is relatively error prone [95], emphasizing the need for experimental validations using techniques such as SHAPE [97], PARIS [100], and SPLASH [101].

## 7. LncRNA Interactions

**Figure 3:**
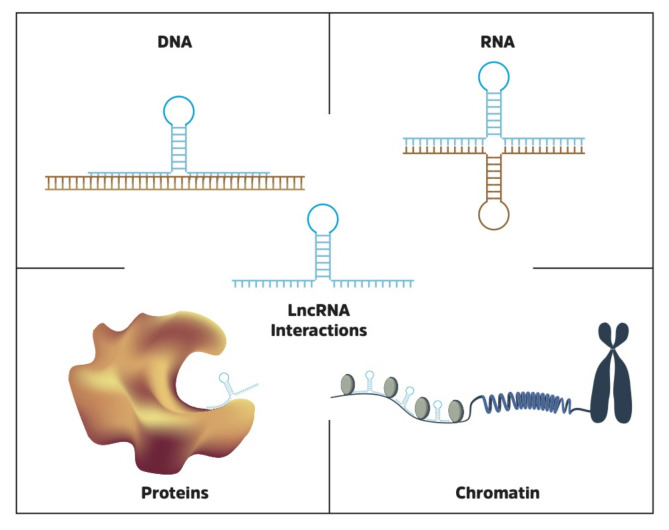
LncRNAs interact with a variety of biological molecules, including DNA, RNA, proteins and chromatin.

Identifying molecular lncRNA interaction partners is critical in the process of understanding function, and thereby their potential impact on cellular processes. For example, the lncRNA *XIST*, which inactivates one of the two X chromosomes in females, requires a physical interaction with Polycomb repressive complex 2 (PRC2) to achieve its function [78]. In addition, the lncRNA *TINCR* is known to control somatic tissue differentiation by interacting with mRNA [118], while the lncRNA *FENDRR* binds directly to DNA to regulate gene expression [119]. The topic of lncRNA functional interactions has recently been reviewed in detail by Marchese et al. [120], and is also discussed by Cech and Steitz [121]. Here, we will focus on databases and tools to identify and characterize RNA, DNA and protein interaction partners of lncRNAs (Figure 3).

### 7.1. RNA Interactome Databases

There are several databases cataloging interactions, as well as numerous prediction tools, which use a variety of methods to predict RNA interactions. Although prediction tools are continuously improving in accuracy, it should be noted that databases containing experimental evidence will be more reliable. Importantly, interaction partners of a lncRNA may also vary in different cell types or in a disease context, depending on the respective transcriptome and proteome of the cell, neither of which are taken into account by prediction algorithms. Here, we discuss two databases of experimentally determined interactions: RNAInter (previously known as RNA Associated Interaction Database (RAID)) [122] and RNA Interactome from Sequencing Experiments (RISE) [123,124], which were chosen based on the breadth of included interactions and ease of use.

#### 7.1.1. RNAInter

RNAInter is designed to be a “one-stop” RNA interactions database [122]. It lists selected interactions of RNAs with proteins, DNA/Chromatin and other RNAs from 35 different databases. The selection comprises targeted as well as high-throughput sequencing experiments, predicted interactions (through miRanda MirTarget and others), and experimental validations of computational predictions [122]. RNAInter was compiled by performing a literature search on over 31,000 published studies, and corresponds to a total of more than 41 million RNA interactions across 154 different species [122]. RNAInter provides an interaction map and a confidence score for the likelihood of the interaction, based on the type of evidence provided [122]. Interactions are automatically ranked by confidence score on a scale of 0 to 1, with 1 being most confident. In addition to experimentally detected interactions, RNAInter has the option to run interaction prediction tools directly from the web server, including IntaRNA, which will be discussed in further detail below [122]. While not being lncRNA-specific, RNAInter contains information for many lncRNAs due to its inclusion of data from various lncRNA-specific databases. Overall, RNAInter is a highly comprehensive database, with an accessible interface that allows for straight-forward in silico investigation of a lncRNA of interest.

#### 7.1.2. RISE

RISE is a database published in 2018, and contains 328,811 RNA-RNA interactions from *H. sapiens*, *M. musculus* and *S. cerevisiae*, as well as from 10 different cell lines across these species [123]. These interactions have been extracted from several high-throughput sequencing experiments [100,101,102,125] and targeted studies, which used methods including Cross-linking, Ligation and Sequencing of Hybrids (CLASH) [126,127], RNA Interactome Analysis and sequencing (RIA-seq) [118] and RNA Antisense Purification (RAP-RNA) [128]. The targeted studies were curated from the RAIN [129], RAIDv2.0 [130], and NPInter [131] databases. For the human interactome, there are 112,444 RNA-RNA interactions, with lncRNA interactions accounting for 20%. Approximately 15,000 of the lncRNA-specific interactions are with mRNAs, with the remaining 7500 interactions spread across miRNAs and a variety of other ncRNA species [123]. RISE compiled many lncRNA interactions, which sets it apart from other databases as a valuable tool. However, it currently lacks a scoring system similar to that of RNAInter [77], providing an estimate of the strength of evidence behind the detected interaction. Overall, RISE is an intuitive database which provides concise information for several lncRNA interactions.

### 7.2. LncRNA-RNA Interaction Prediction

Tools to predict RNA-RNA interactions have been available since the late 2000 s, with the number of tools released continuously increasing. Although these algorithms are useful in the absence of experimental evidence for a lncRNA of interest, they often result in low prediction accuracy when benchmarked [107,108], similar to many other in silico prediction approaches. The majority of RNA-RNA interaction prediction tools use the thermodynamic principle of finding the MFE of the interaction [108]. Other methods include alignment-based approaches such as RIsearch [132], homology based methods including PETcofold [133], and deep learning models such as GPLPI [134]. Here, we discuss two tools to analyze RNA-RNA interaction potential. The first tool, IntaRNA [135], uses MFE principles, and takes into account sequence accessibility, equating to both the binding energy and the unbinding (opening) energy of the two sequences being assessed. The second tool, LncRRIsearch [136] is a web server integrating the methodology of the authors’ previously released command-line tool, RIblast [137] (based on interaction energy that is computed by using both accessibility energy and hybridization energy) with tissue-specific expression and subcellular localization data to improve prediction accuracy [138]. IntaRNA requires prior knowledge of sequence information for both interaction partners, making it more suited for the prediction of specific targeted interactions. On the other hand, LncRRIsearch requires only one query lncRNA, making it more useful for lncRNA interaction discovery.

#### 7.2.1. IntaRNA

IntaRNA 2.0 [135] is an open-source tool developed as part of the Freiburg RNA Tools suite [139]. It was reimplemented in 2017 based on the highly popular IntaRNA 1.0, which was originally published in 2008 [140]. IntaRNA 1.0 performed in the top three of tools benchmarked by Umu and Gardner [108], receiving a Matthews correlation coefficient (MCC) of 0.58 (on a scale of −1 to 1, where 1 is a perfect tool). IntaRNA 2.0 has not yet been benchmarked against other similar tools. IntaRNA 2.0 makes predictions on the likelihood of RNA-RNA interactions, based on MFE and sequence accessibility, and uses seed stability constraints, providing further reproducibility to data outputs [135]. The algorithm also takes into account energy contributions of “dangling end” base pairs and investigates suboptimal interaction alternatives [135]. IntaRNA 2.0 has an easily accessible web server available, using FASTA formatted sequences as input [135]. Outputs are available in both tabulated formats and dot-bracket output alongside a visual representation. IntaRNA is likely to be most useful for those investigating whether a novel lncRNA may interact with previously identified genes or transcripts of interest in a low-throughput context, and where experimental information is not available.

#### 7.2.2. LncRRIsearch

LncRRIsearch is a web server for rapid identification of lncRNA-mRNA and lncRNA-lncRNA interactions in human and mouse [136], and differs from IntaRNA in several areas. First, in order to increase the accuracy of prediction, subcellular localization data from lncATLAS [76] and tissue-specific expression data from five human [21,141,142,143,144] and four mouse RNA-seq datasets [145,146] have been included in the prediction algorithm. Secondly, while it still uses principles of MFE estimation and accessibility, the integrated prediction tool RIblast [137] reduces prediction times by using a heuristic seed search and extension approach. This method identifies short seed regions in all interactions compared to all possible interactions, and scores according to length and hybridization energy, prior to putative interactions being extended from each end of the seed. If the interaction energy exceeds the set threshold energy, the seed extension is terminated. LncRRIsearch has used this method to pre-calculate comprehensive human and mouse lncRNA interactomes, which are stored in a MySQL database. This system is similar to that used by BLAST to ensure results are provided rapidly to the user [50]. LncRRIsearch also allows lncRNA query searches without specifying interaction partners, meaning that users can get an estimation of possible interacting RNA sequences without prior knowledge of what these may be.

### 7.3. LncRNA-DNA Interaction

Investigation of lncRNA-DNA interactions have the potential to elucidate lncRNA-mediated regulation of gene expression. This is exemplified by the recruitment of chromatin modifying enzymes by the lncRNA *HOTAIR*, resulting in histone modifications [147], and the direct binding of DNA promoter elements by the lncRNA *FENDRR*, forming a lncRNA-DNA triplex resulting in recruitment of the PRC2 complex [119]. The field of lncRNA-DNA interactions has been reviewed in detail by Rinn and Chang (2012) [148], and discussed more recently by Marchese et al., (2017) [120]. Here, we will distinguish between direct lncRNA-DNA interactions and more general lncRNA-chromatin interactions, discussing LnChrom [149], an experimentally validated database of lncRNA-chromatin interactions, and Triplexator [150], a popular command-line tool for predicting lncRNA interactions directly with DNA.

#### 7.3.1. LnChrom

Although the existence of lncRNA-chromatin interactions has been established for several years, development of databases and tools for investigation of such interactions is still an emerging field. LnChrom is a recently published, comprehensive database of experimentally validated lncRNA-chromatin interactions [149]. Although 138,062 RNA-DNA interactions from four datasets [151,152,153,154] compiled in LnChrom are included in the RNAInter database [122] discussed earlier, this makes up only one third of the available data in LnChrom, meriting the LnChrom database further discussion here. The aim of LnChrom is to elucidate regulatory mechanisms of lncRNAs [149]. It contains expertly curated information on 382,743 experimentally detected lncRNA-chromatin interactions, involving 2390 lncRNAs across 263 human and mouse tissue types. It also includes multi-omic and metadata on each interaction pair, such as chromatin modifications, associated proteins, and any diseases associated with lncRNA-mediated chromatin regulation. The majority of the information in the database is from high-throughput experiments such as ChIRP-seq [151] and CHART-seq [155], and 70% of the total data is categorized as human. To compile LnChrom into a functional and broad dataset, the authors inquired PubMed for interaction information using keywords, yielding 8000 papers, and searched the NCBI GEO datasets [156] for interaction pairs detected using high-throughput experiments. The combined search results were filtered to a total of 27 high quality datasets, which were processed to retrieve lncRNA binding site information according to their standard pipeline, described in detail in Chu et al., 2011 [151]. LnChrom provides a genome browser for visualization of interactions, accompanied by outsourced expression data [157], transcription factor occupancy data [158] and a cancer exploration panel [42,43]. Although not all interactions are associated with additional information, LnChrom provides references to the original publication, and/or further literature to validate the interaction when available. Overall, LnChrom is user-friendly, easily searchable and provides useful information on many lncRNA-chromatin interactions. LnChrom will be constantly improving as the authors aim to continue updating the database as new datasets become available, with the goal of generating a supervised interaction prediction tool in the future [149].

#### 7.3.2. Triplexator

One method of predicting a direct lncRNA-DNA interaction is to estimate the likelihood of triplex formation, where a single stranded RNA undergoes Hoogsteen base-pairing with the double stranded DNA, forming a three stranded structure [159,160]. In the absence of experimental evidence for the formation of such a triplex by a lncRNA of interest, there are a limited number of prediction algorithms available to assess the likelihood of lncRNA-DNA triplex formation. These have been discussed in a benchmark by Antonov et al., in 2018 [161], suggesting Triplexator [150] as the most accurate and usable tool currently available. Triplexator is a brute force prediction algorithm for the identification of RNA-DNA triplexes in *H. sapiens*, *M. musculus*, *D. rerio*, *D. melanogaster* and *C. elegans* [150]. Brute force algorithms rely on compute power to conduct exhaustive searches of all possible options in any given query, as described by Mohammad et al. [162]. Triplexator uses the same methodology alongside a q-gram-based filter, which discards sequence regions that do not meet triplex formation criteria prior to testing all remaining options. The algorithm works under the assumption that triplexes are sufficiently modeled by the canonical binding rules of Hoogsteen and reverse Hoogsteen nucleotide triad formation [150]. First, it identifies sequence features of a query lncRNA that has the potential to bind to a DNA target site. In the absence of a specified target sequence, Triplexator then scans a q-gram filtered reference genome (hg19 for human targets) for putative binding sites [150]. The user can specify the minimum and maximum length of a triplex interaction, the number of errors allowed (with more errors usually being allowed in longer triplexes, due to their increased stability [150]), and the minimum guanine content allowed for a triplex. While being a very useful tool for the scientific community, Triplexator could be improved in two ways. When benchmarked by Antonov et al. [161], Triplexator performed with an Area Under the Curve (AUC) value of 0.61, leaving some room for optimization of the algorithm, perhaps by taking into account chromatin accessibility of target regions. Secondly, the genomes used to scan for target DNA sites could be updated to the most recent annotations.

### 7.4. LncRNA-Protein Interaction Prediction

LncRNA interactions with proteins have been shown to drive important cellular processes such as recruitment of protein complexes to chromatin [163,164] as well as post-transcriptional regulation of gene expression, splicing and translation [8]. There is an abundance of tools to predict lncRNA-protein interactions, and these have been reviewed in great depth by Peng et al., (2020) [165]. Here, we will discuss a machine-learning, ensemble-based method called SFPEL-LPI (sequence-based feature projection ensemble learning method–lncRNA-protein interaction) [166]. It had the highest performance score when benchmarked by Peng et al., with an AUC value of 0.97 in their leave-one-out cross validation, and of 0.92 on a five-fold cross validation.

#### SFPEL-LPI

SFPEL-LPI is a prediction algorithm that finds known and predicted interactions between RNAs and proteins by using a feature projection ensemble learning frame to integrate sequence derived features and similarities [166]. The SFPEL-LPI algorithm comprises several steps. Based on a lncRNA NONCODE ID [4] query, it first downloads known lncRNA-protein interactions from NPInter [131], lncRNA sequences from NONCODE [4] and finally, protein sequences from SUPERFAMILY [167]. Next, it describes query features based on their dinucleotide or amino acid composition for the lncRNA and query protein, respectively. More specifically, this is known as parallel correlation pseudo dinucleotide or amino acid composition. SFPEL-LPI then assesses potential interaction partner features in the same way, according to their dinucleotide or amino acid composition. Finally, it compiles these into a features matrix, and compares their similarity, producing association scores for potential lncRNA-protein interactions. SFPEL-LPI outputs a downloadable list of experimentally validated and predicted interactions, which can be filtered by association score and visualized as a network. The association score is based on the Smith Waterman algorithm for calculating similarity of biological sequences, on a scale of 0 to 1, with 1 being an experimentally validated protein interaction. The network visualization color codes the known and predicted interactions, and provides the top gene ontology (GO) terms associated with each. SFPEL-LPI is a highly accurate and user-friendly tool, which generates important information about the possible function of a lncRNA of interest.

The myriad of possible interaction partners makes lncRNA interaction databases and predictive tools a complex and emerging field of research. Understanding the interactions of lncRNAs is an important starting point towards elucidating their potential functions. Additional tools take it one step further, directly providing functional prediction of lncRNAs, as reviewed below.

## 8. Function Prediction

Predicting the function of a lncRNA from sequence alone has been a challenging task in the field of lncRNA biology [168,169]. Here we describe a predictive tool, SEEKR, which uses k-mer-based classification to compare lncRNAs and infer their function.

### SEEKR

LncRNAs which broadly serve similar functions, for example *Xist* and *Kcnq1ot1i*, which both regulate gene expression in cis through the PRC, often have little to no sequence similarity. Accordingly, discerning the function of one lncRNA experimentally likely does not yield insights into the functions of other lncRNAs. To overcome this, Kirk et al. developed SEEKR (Sequence Evaluation from K-mer Representation), which uses the relative frequencies of k-mers in lncRNA sequences to infer function based on similarity to other lncRNAs [168]. SEEKR is designed to count the appearance of k-mers of specified lengths along the sequence of a lncRNA, and normalize these counts to develop a “k-mer profile” [168]. The k-mer profiles for two lncRNAs can be tested for similarity using a Pearson correlation, allowing for two lncRNAs which would share little linear homology to have their k-mer profile similarities brought to light [168]. In practice, this would guide a researcher on experimental methodologies to validate the function of a lncRNA if it shows high k-mer similarity to a well-characterized lncRNA.

SEEKR can be used to infer the function of a lncRNA of interest, providing researchers with a useful starting point for further functional characterization in the lab. Examples for experimental validation include the generation and in vitro characterization of knockout/knock-in/knockdown models coupled to RNA-Seq, mass spectrometry, and/or metabolic assays [170,171]. A problem with several published lncRNA functional prediction tools, with citations in the hundreds, is that they are occasionally not maintained reliably or cease to be available altogether.

## 9. Conclusions

With rapidly increasing interest in lncRNAs and their widespread roles across disease, genomic regulation and development, databases and prediction tools are essential as a first step in characterizing a new lncRNA. For example, if the LncATLAS database shows a lncRNA localizes to the nucleus, and Triplexator predicts that it can form a triplex with a DNA sequence, the next step of characterization could be a ChIRP experiment, to determine which particular regions of DNA the lncRNA is interacting with.

Future directions in the field are wide-ranging. Subcellular localization prediction is still an emerging field with few tools at present. As more experimental localization data becomes available, prediction algorithms should improve in accuracy, due to larger and more accurate training and benchmarking datasets, in addition to more robust algorithm design platforms becoming available. Although there are many programs to predict RNA interactions and structures, these often struggle with longer or more complex structures, especially those containing pseudoknots. This may be a long-term weakness due to the complexity of pseudoknots; however, some newer tools are beginning to touch on the area (for example, DMfold [96,172]). Although there is high-throughput information available for many lncRNA-chromatin interactions such as that stored in LnChrom [149], tools to predict RNA-chromatin interactions that have not been experimentally detected are very limited in number, and those that are available do not have a high level of usability for a less specialized audience. Development of an easy-to-use web server could be an improvement for this area. One fascinating aspect of predictive algorithms is the implementation of neural networks over older machine learning methodologies. One predictive tool covered here (LncLocator) uses neural networks; however, this is only the beginning of more advanced artificial intelligence methodologies being applied to lncRNA research.

As more tools and databases are released each year, keeping track of the most accurate and expansive of each will be important for lncRNA researchers. As such, annual benchmarks of tools and reviews of databases would be helpful. What could aid in the benchmarking of existing tools, and in the training of new tools, is the construction of a ‘master lncRNA training database’. This would consist of many well-characterized and thoroughly experimentally validated lncRNAs, which would include localization data, known interactions, disease associations, and expression levels across tissue types/species.

## Appendix A

**Table A1:** List of tools and databases reviewed.

Tool/Database Name	Description	Link	Citation
General lncRNA Databases			
LNCipedia	General lncRNA database, expertly curated	https://lncipedia.org/	[18,19]
LNCBook	General lncRNA database, some community curation	http://bigd.big.ac.cn/lncbook/index	[29]
Expression Databases			
GTEx	Comprehensive public resource to study tissue-specific gene expression and regulation.	https://gtexportal.org/home/	[39,40]
TANRIC	Database of non-coding RNAs in cancer	www.tanric.org	[42]
CANTATAdb	Database of plant lncRNAs	http://cantata.amu.edu.pl/	[48,49]
Protein Coding Potential			
CPC	Protein coding potential prediction	http://cpc.gao-lab.org/	[55]
CPPred	Protein coding potential prediction	http://www.rnabinding.com/CPPred/	[60]
CNIT	Protein coding potential prediction	http://cnit.noncode.org/CNIT/	[62]
Subcellular Localization			
LncSLdb	Database of lncRNA subcellular localization	http://bioinformatics.xidian.edu.cn/lncSLdb/	[72]
LncATLAS	Database of lncRNA subcellular localization	https://lncatlas.crg.eu/	[76]
LncLocator	LncRNA subcellular localization prediction	http://www.csbio.sjtu.edu.cn/bioinf/lncLocator/	[77]
Structural Conformation			
RMDB	Database of RNA structures	https://rmdb.stanford.edu	[94]
RNAfold	RNA structure prediction	http://rna.tbi.univie.ac.at/, http://rna.tbi.univie.ac.at/cgi-bin/RNAWebSuite/RNAfold.cgi	[95]
DMfold	RNA structure prediction with pseudoknots	https://github.com/linyuwangPHD/RNA-Secondary-Structure-Database	[96]
Interactions			
RNAInter	Database of RNA interactions	http://www.rna-society.org/rnainter/	[130]
RISE	Database of RNA interactions	http://rise.life.tsinghua.edu.cn	[123]
IntaRNA 2.0	RNA-RNA interaction prediction	http://rna.informatik.uni-freiburg.de/IntaRNA/Input.jsp	[135]
LncRRISearch	LncRNA-RNA interaction prediction	http://rtools.cbrc.jp/LncRRIsearch/	[137]
LnChrom	Database of lncRNA-Chromatin interactions	http://biocc.hrbmu.edu.cn/LnChrom/	[149]
Triplexator	RNA-DNA interaction prediction	http://bioinformatics.org.au/tools/triplexator/	[157]
SFPEL-LPI	LncRNA-protein interaction prediction	http://bioinfotech.cn/SFPEL-LPI/	[166]
Function Prediction			
SEEKR	K-mer similarity predictor	http://seekr.org/home	[168]
